# Enhancing immune resilience in swine: a comprehensive meta-review of preventive and host-directed strategies against African swine fever and swine influenza

**DOI:** 10.3389/fvets.2026.1919982

**Published:** 2026-09-22

**Authors:** Mike K. S. Chan, Thomas Skutella, Fiona Macniesia Thomas, Jonathan Robert Todd Lakey, Adrian Mutto, Katsuhiko Fukuda, Liu Baochi, Michelle B. F. Wong

**Affiliations:** 1 European Wellness Academy, Frankfurt, Germany; 2 European Wellness Biomedical Group, Frankfurt, Germany; 3 Baden Research & Testing Laboratories, Kota Kinabalu, Sabah, Malaysia; 4 Lincoln University College, Petaling Jaya, Selangor, Malaysia; 5 Institute for Anatomy and Cell Biology, Heidelberg University Medical Faculty, Heidelberg, Germany; 6 Department of Surgery, University of California, Irvine, Irvine, CA, United States; 7 Instituto de investigaciones Biotecnológicas, UNSAM-CONICET, Buenos Aires, Argentina; 8 Integrated Medical Centre Fukuda, Matsue, Shimane, Japan; 9 Shanghai Public Health Clinical Centre, Fudan University, Shanghai, China; 10 FCTI R&D GmbH, Edenkoben, Germany

**Keywords:** African swine fever, biosecurity, herd management, host-directed therapy, swine influenza, transboundary

## Abstract

African swine fever (ASF) and swine influenza (SI) are among the most consequential infectious threats to global swine production because they combine major economic burden with fundamentally different transmission ecology, tissue tropism, and immune-pathological behavior. ASF is a highly lethal systemic disease characterized by macrophage tropism, immune dysregulation, environmental persistence, and severe trade and production consequences, whereas SI is a highly transmissible respiratory infection associated with recurrent herd-level morbidity, viral evolution, and zoonotic significance. Although these diseases differ markedly in biology and field presentation, both highlight the limitations of isolated pathogen-centered control strategies when implemented without integrated herd-level resilience measures. This meta-review synthesizes the cited literature into an evidence-based framework encompassing epidemiology, pathogenesis, surveillance, vaccination, biosecurity, herd management, supportive care, nutritional and immune-supportive measures, and host-directed regenerative approaches. Across the reviewed literature, the strongest field-supported evidence remains centered on surveillance, vaccination where applicable, biosecurity, and production-system management. At the same time, regenerative and host-supportive biologic strategies, including mitochondria-targeted peptides, thymic and organ-derived peptides, extracellular vesicle-based approaches, and stem cell-related therapies, demonstrate growing translational and immunomodulatory relevance within the broader context of immune resilience and recovery support. Comparative analysis further demonstrates that intervention priorities must remain aligned with disease biology: ASF control depends primarily on preventing viral introduction and limiting outbreak dissemination, whereas SI management benefits from integrated vaccination strategies, environmental optimization, mitigation of secondary bacterial disease, and continual adaptation to antigenic evolution. Within this framework, immune resilience is best understood not as a replacement for conventional disease control, but as a layered outcome arising from reduced infection pressure, preserved immune competence, regulated inflammatory responses, tissue recovery capacity, and supportive production practices.

## 1 Introduction

Pork remains one of the most widely consumed animal proteins worldwide, making swine health central to food security, farm income, and stability of international agricultural trade (1). In parallel with rising demand, pig production systems have become more intensified, more interconnected, and in many regions more vulnerable to infectious disease due to dense animal populations, movement networks, and uneven biosecurity infrastructure. Viral diseases are therefore no longer only clinical problems at farm level; they are increasingly structural threats to productivity, market access, and regional food systems (2, 3).

Among these threats, African swine fever and swine influenza occupy particularly important positions because they represent two distinct but complementary stress tests of modern swine systems. ASF is caused by African swine fever virus (ASFV), a large double-stranded DNA virus. Its severity depends on the interplay between viral strain virulence and host immune responses, as reflected in variations in clinical signs and mortality across diverse regional epidemiological scenarios (4). In contrast, SI is caused primarily by influenza A viruses (IAVs), which are segmented RNA viruses characterized by substantial genetic diversity and zoonotic potential (5). Clinically, SI is a highly transmissible respiratory disease associated with high morbidity, depressed growth performance, risk of secondary bacterial infection, and ongoing productivity loss at herd level (6). ASF, by contrast, is a severe systemic viral disease with mortality that can approach 100% in susceptible populations and remains defined by the absence of widely deployable curative treatment, the importance of environmental persistence, and the necessity of strict outbreak control measures (7, 8).

A side-by-side analysis of ASF and SI is analytically useful because these diseases reveal different points of fragility in swine production. SI illustrates the burden of endemic circulation, antigenic evolution, and repeated respiratory outbreaks, whereas ASF illustrates the catastrophic consequences of a high-fatality transboundary disease in which prevention of entry is more feasible than therapy after establishment (9). Accordingly, this review adopts a meta-analytic perspective, with stronger emphasis on evidence hierarchy, biological plausibility, practical readiness, and the distinction between established intervention pillars and emerging approaches.

## 2 Scope and meta-review approach

A key challenge in the literature is that interventions supported by strong field evidence are often presented alongside emerging biologic or regenerative concepts with evidence bases at molecular level, and without sufficiently clear stratification by evidentiary strength or practical relevance specifically in pig. That concern is particularly important in veterinary infectious disease because the practical usefulness of an intervention depends not only on mechanistic plausibility but also on cost, scalability, route of administration, herd-level logistics, and compatibility with commercial production (1, 10).

In this meta-review, the literature is interpreted through four evidence layers. The first includes established field interventions such as biosecurity, surveillance, herd management, and commercially used SI vaccines (6, 11, 12). The second includes validated but context-dependent tools, such as emerging ASF vaccines that demonstrate strong pig challenge data but still face deployment and regulatory considerations (13, 14). Despite advances in ASF vaccine development and the authorization of some vaccine candidates for use in parts of Asia, there is currently no licensed ASF vaccine in the European Union. The third includes biologically grounded approaches supported by experimental and translational pig studies, including β-defensin-related strategies and other immune-modulating interventions (15, 16). The fourth includes host-directed and regenerative approaches, encompassing peptide-based, cellular, and signaling-modulation strategies that aim to enhance immune competence, tissue repair, and overall resilience under infectious challenge (17–19).

For the present analysis, immune resilience is defined as the capacity of pigs and herds to limit infection, reduce disease severity, preserve immune competence, maintain tissue integrity, and recover functional productivity under pathogen exposure (20, 21). This definition deliberately avoids vague notions of generalized immune stimulation and instead emphasizes an interaction among pathogen pressure, innate and adaptive immune quality, inflammatory regulation, environmental load, and management structure. Within this framework, resilience is not a single therapy but an emergent outcome of layered control.

## 3 Epidemiology and global disease burden

African swine fever (ASF) and swine influenza (SI) are distinct infectious disease with markedly different epidemiological characteristics and represent major threats to the global porcine sector. ASFV and swine influenza A viruses differ profoundly in how they persist and spread across swine populations. ASFV persistence is shaped by environmental stability, contamination of fomites and pork products, wildlife reservoirs or interfaces, and human-mediated dissemination events, including long-distance jumps linked to transport and biosecurity breaches (22–25). In contrast, SI persists through endemic respiratory transmission, ongoing reassortment, antigenic drift, and repeated movement of viruses through swine populations interconnected by trade and production systems (26–29).

These contrasting ecologies have practical consequences. ASF often behaves as a transboundary emergency in which the central question is whether the virus can be excluded or rapidly contained after entry (4, 30). SI behaves more like a constantly shifting endemic respiratory threat that repeatedly erodes production through cycles of infection, reinfection, and antigenic mismatch (6, 31). Both are damaging, but they operate on different temporal and epidemiological scales (Figure 1).

**Figure 1:**
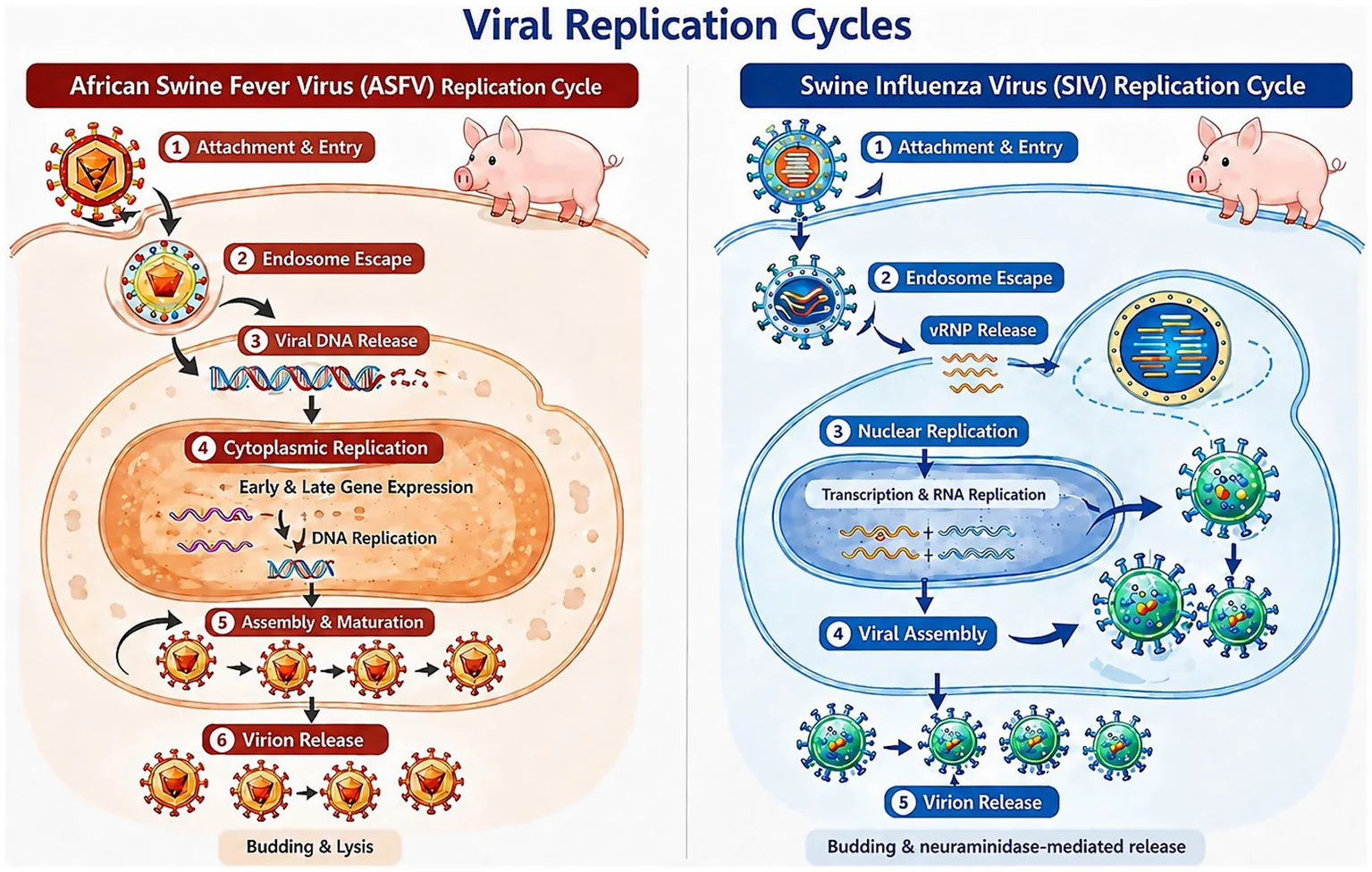
Comparative replication cycles of African swine fever virus (ASFV) and swine influenza virus (SIV). ASFV infects macrophages and replicates predominantly in the cytoplasm within perinuclear viral factories following endosomal escape and viral DNA release. Early and late gene expressions precede genome replication, virion assembly, maturation, and release via budding or cell lysis. In contrast, SIV infects respiratory epithelial cells and undergoes endocytosis followed by hemagglutinin-mediated membrane fusion and uncoating. Viral ribonucleoproteins are transported to the nucleus, where transcription and RNA replication occur, and progeny virions assemble at the plasma membrane and are released by neuraminidase-facilitated budding.

ASF spread since the emergence of the genotype II pandemic lineage has been characterized by persistent regional expansion, wildlife-associated maintenance, and episodic jump events across affected territories (4, 25, 32). However, ASFV exhibits substantial genetic diversity, with multiple genotypes circulating in Africa, while genotypes I and II have achieved broader geographical spread beyond the continent. Genotype I ASFVs were detected in domestic pigs in China in 2021, including low-virulence but efficiently transmissible strains associated with chronic infection (33). More recently, naturally occurring genotype I/II recombinant ASFVs have also been identified, including highly virulent and transmissible recombinant viruses detected in China (34). The co-circulation and recombination of genetically distinct ASFV lineages add further complexity to surveillance, disease control, and vaccine development. Consequently, this genetic diversity may impact vaccine efficacy, as cross-protection against heterologous ASFV strains and genotypes can be variable (35). Rathakrishnan et al. (36) demonstrated that a gene-modified genotype II live attenuated vaccine conferred complete homologous protection against genotype II ASFV, but only partial protection against genotype I and no protection against genotype IX, reflecting the need to consider circulating genotype diversity when evaluating the geographical applicability and breadth of ASF vaccines. In contrast, SI circulation is globally interconnected yet regionally structured, with North America and Europe acting as important source regions in long-term phylogeographic analyses, and live swine movement serving as a major driver of dissemination (5, 29, 37). The emergence of A(H1N1)pdm09 in 2009 illustrates the potential contribution of swine-origin influenza lineages to pandemic emergence. Following human-to-swine transmission, A(H1N1)pdm09 viruses can persist, evolve, and reassort with endemic swine influenza viruses, contributing to viral diversity and subsequent zoonotic risk (38). Markin et al. (39) and Zanella et al. (40) further demonstrated that continued circulation and evolution of these viruses in swine can increase genetic and antigenic diversity and maintain opportunities for subsequent zoonotic transmission. These patterns indicate that control strategies cannot simply transfer from one disease to the other; intervention design must reflect whether persistence is driven mainly by environmental durability and introduction events, or by respiratory circulation and viral evolution. For both diseases, current viral diversity emphasizes the importance of surveillance to detect newly emerging genotypes, reassortants, and variants that could alter transmission, vaccine efficacy, or zoonotic risk.

## 4 Transmission ecology and surveillance constraints

Transmission ecology sits at the interface between pathogen biology and farm practice. In ASF, circulation may involve domestic pigs, contaminated pork products, fomites, wild boar, and in some ecological context soft ticks (8, 22, 24). The importance of environmental survival means that virus introduction can occur even without direct pig-to-pig contact, which is why disinfection, feed safety, carcass management, and control of human and vehicle traffic are so important (11, 12, 23).

In SI, by contrast, aerosol and direct-contact transmission dominate (41). High animal density, continuous flow systems, mixing of age groups, and repeated entry of new animals all favor persistence and spread (1, 37). Because influenza A viruses also reassort and accumulate antigenic changes, the epidemiological problem is not just the occurrence of outbreaks but the continual generation and circulation of immunologically variable viruses (26, 28).

Surveillance limitations affect both diseases but in different ways. For ASF, variable reporting timeliness, inconsistent premise classification, and structural data gaps impede rapid detection and cross-country interpretation of outbreak patterns (32, 42). For SI, voluntary sampling systems, asymptomatic infections, and incomplete surveillance at the animal-human interface can underestimate prevalence and obscure zoonotic risk (43, 44). These weaknesses are not merely technical; they determine whether interventions are applied early enough to matter.

## 5 Comparative pathogenesis and control implications

ASF is caused by a large double-stranded DNA virus with marked macrophage tropism, extensive immune evasion capacity, and severe systemic effects including vascular injury, coagulopathy, and lymphoid depletion (8, 45, 46). The pathogenesis of ASFV is primarily driven by viral replication in monocytes and macrophages within lymphoid tissues, including the spleen, lymph nodes, and tonsils (46). Following entry via endocytosis or micropinocytosis, the virus replicates in the cytoplasm and expresses immunomodulatory proteins that disrupt interferon signaling and cytokine responses, thereby leading to systemic dissemination and profound immune dysfunction. In parallel with its suppression of effective antiviral immunity, dysregulated activation of infected macrophages and excessive production of pro-inflammatory mediators may contribute to systemic inflammation, which promotes vascular damage and tissue injury during severe ASFV infection. The pathological characteristics of ASFV infection can be categorized into (a) acute ASFV, characterized by petechial hemorrhages and splenomegaly, (b) subacute ASFV, such as intense edema and bleeding, pericardial effusion and frequent splenomegaly and (c) terminal stages characterized by pulmonary edema leading to respiratory distress and death due to circulatory shock (14). SI, in contrast, is caused by a segmented RNA virus that replicates in respiratory epithelial cells and escapes host immunity primarily through antigenic drift and reassortment rather than through the same degree of intracellular immune suppression (6, 28, 47). Its genome consists of eight segmented RNA molecules that undergo antigenic drift through the accumulation of mutations, while reassortment between influenza viruses can result in antigenic shift. These segments encode the basic polymerase proteins 1 and 2 (PB1 and PB2), the acidic polymerase protein (PA), hemagglutinin (HA), nucleoprotein (NP), neuraminidase (NA), matrix proteins (M1 and M2), and nonstructural proteins, including NS1 (48). HA and NA are the two major surface glycoproteins; HA mediates receptor binding and membrane fusion, whereas NA facilitates the release of newly formed virions from infected cells by cleaving sialic acid residues. To initiate infection, IAVs primarily target epithelial cells of the upper and lower respiratory tract through HA-mediated binding to sialic acid receptors, followed by endocytosis, endosomal acidification-triggered membrane fusion, and nuclear import of viral ribonucleoprotein complexes (vRNPs) for replication (49, 50). This viral replication causes epithelial cell necrosis, impairs mucociliary function, and activates innate immune responses, including the production of type I interferons and pro-inflammatory cytokines (51). IAV infection typically presents as an acute respiratory disease with a rapid onset of coughing, nasal discharge, fever, and lethargy, while severe infections may be associated with conjunctivitis, dyspnea, and tachypnea (51). These differences in viral pathogenesis and host responses are central to intervention logic.

ASFV interferes with type I interferon signaling, impairs antigen presentation, and promotes immune dysfunction that complicates vaccine design and limits reliable natural protective immunity (14, 45). As monocytes and macrophages are the primary target cells of ASFV, infection can alter macrophage-mediated innate antiviral responses, including interferon signaling and antigen-processing function. These virus-host interactions reflect the relevance of macrophage-based models, including iPSC-derived macrophages, for investigating ASFV infection and immune evasion. In addition, African wild suids, particularly warthogs and bushpigs, exhibit natural resistance to the pathogenic effects of ASFV, as they can become infected while remaining clinically healthy (52). This asymptomatic phenotype reflects disease tolerance rather than complete resistance to infection, as these animals can support viral replication while remaining subclinical. However, the precise mechanisms underlying this tolerance have yet to be fully elucidated. Differences in innate responses, particularly species-specific macrophage responses, may partly explain this phenotype. Recent comparative work showed that warthog-derived macrophages supported ASFV infection without inducing interferon-alpha (IFN-α), unlike macrophages derived from the other suids examined (53). Notably, whether this cellular difference contributes to disease tolerance *in vivo* remains to be established. In contrast, immune defences against SI infection are primarily initiated within the respiratory tract and are characterized by local interferon and inflammatory cytokine release, systemic and mucosal antibody responses, and virus-specific CD4 + and CD8 + T-cell activation. However, the protective value of these responses is undermined by viral evolution and imperfect antigenic matching (54–56). In practical terms, ASF control emphasizes exclusion, containment, and highly cautious vaccine development, whereas SI control emphasizes maintaining respiratory herd health and updating immune protection in the face of viral diversity (Table 1).

**Table 1:** Comparative pathogenetic and control-relevant features of ASF and SI.

Feature	African swine fever	Swine influenza	Implication for control
Viral genome	Large dsDNA virus	Segmented ssRNA negative-sense virus	ASF involves complex immune evasion; SI evolves rapidly through mutation and reassortment
Primary target cells	Monocytes/macrophages	Respiratory epithelial cells	ASF is systemic and strongly immune-disruptive; SI is mainly respiratory and highly transmissible
Replication site	Cytoplasmic viral factories with limited nuclear involvement	Nuclear transcription and RNA replication	Different antiviral vulnerabilities and vaccine challenges
Dominant immune challenge	Interferon suppression, impaired antigen presentation, lymphoid depletion	Antigenic drift, reassortment, and variable vaccine match	ASF control depends on prevention and containment; SI control depends on adaptable vaccination and herd-level respiratory management
Clinical pattern	Acute systemic hemorrhagic disease	Acute respiratory disease with high morbidity and lower mortality	Different priorities for therapy, prevention, and surveillance
Mortality	High, often approaching 100% in acute outbreaks	Usually low, though coinfections can worsen outcome	ASF is a transboundary emergency; SI is a recurrent endemic productivity disease

## 6 Vaccination as the leading biologic strategy

Vaccination remains the most mature biologic intervention class across the literature considered here, but the contrast between ASF and SI is striking. For ASF, multiple platforms are under investigation, including gene-deleted live attenuated viruses, naturally attenuated strains, subunit vaccines, viral-vectored constructs, DNA vaccines, and epitope-focused approaches (8, 13, 14). The current evidence, strengths, and limitations of these different vaccine platforms are summarized in Table 2. Naturally attenuated strains have demonstrated varying degrees of protection but may raise concerns about residual virulence and the consistency of protection. Subunit, DNA, viral-vectored, and epitope-based vaccines provide safer and more controlled approaches; however, their protective efficacy has generally been variable, reflecting challenges in identifying the viral antigens and immune responses needed for effective protection (14, 57, 58). Among these, gene-deleted live attenuated ASF vaccines currently provide the strongest experimental evidence in pigs, with several candidates demonstrating substantial protection against homologous challenge (Figure 2). ASFV-G-ΔI177L is a gene-deleted live attenuated vaccine developed by deleting the I177L gene from the virulent ASFV Georgia strain. In the initial study, groups of five pigs vaccinated with different doses of ASFV-G-ΔI177L remained clinically normal and were protected against challenge with the parental ASFV-G strain (59). A subsequent study further investigated the cellular immune response to ASFV-G-ΔI177L in groups of 10–12 pigs and demonstrated changes in CD4+, CD8+, and CD4 + CD8 + memory T-cell populations following vaccination (60).

**Table 2:** Vaccine platforms discussed in the literature.

Platform	Target disease	Evidence status	Main strength	Main limitation
Gene-deleted live attenuated vaccine	ASF	Strong pig challenge data	High homologous protection	Safety, breadth, regulation, and deployment concerns
Naturally attenuated strains	ASF	Historical and moderate evidence	Reduced virulence compared with parent strains	Limited and inconsistent protection
Subunit / epitope / DNA / vectored vaccines	ASF	Experimental	Safer conceptual platforms	Lower or more variable protection than leading LAV approaches
Whole inactivated vaccines	SI	Established field use	Reduced disease severity and viral shedding with antigenic match	Limited cross-protection and mismatch problems
LAIV / replicon / vectored SI vaccines	SI	Emerging experimental and developmental literature	Potential for broader cellular and mucosal immunity	Field maturity still variable

**Figure 2:**
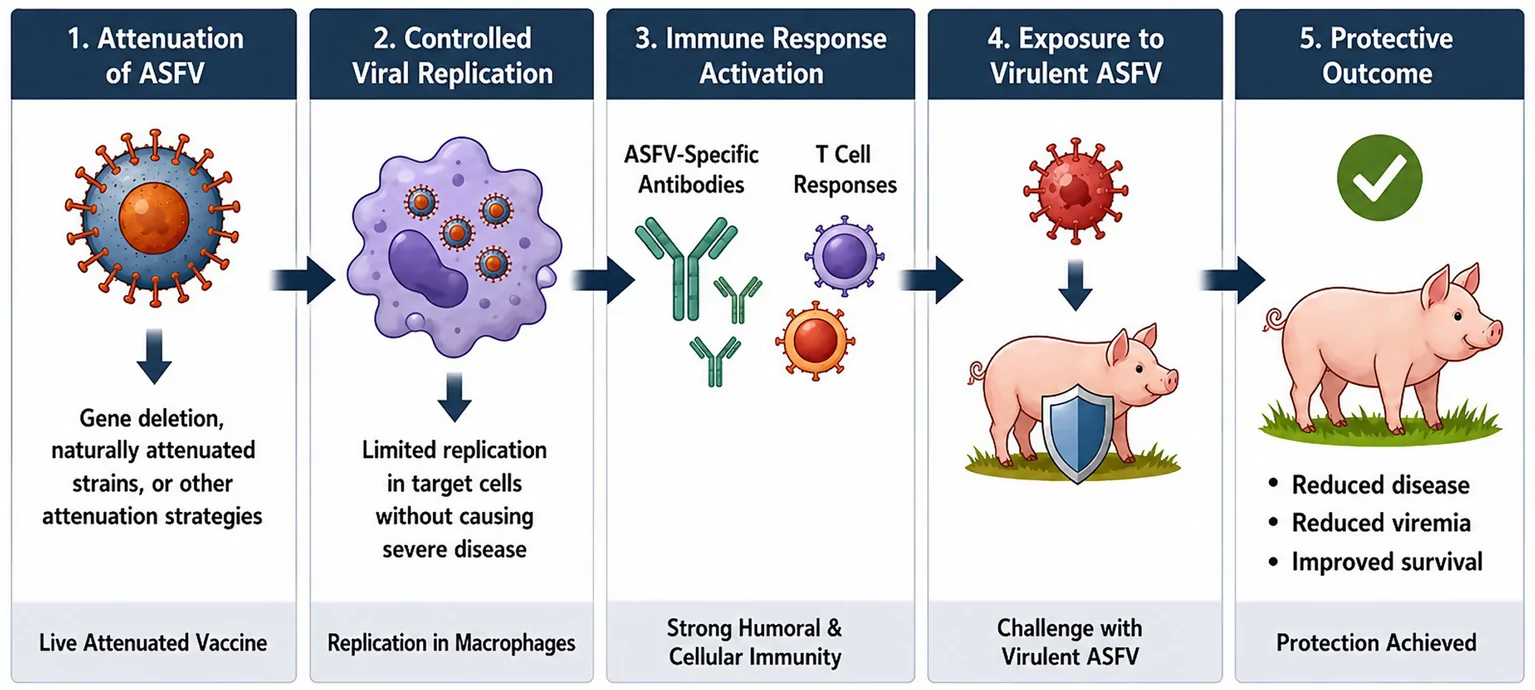
Schematic representation of protective responses associated with live attenuated African swine fever virus (ASFV) vaccines. Attenuation of ASFV through gene deletion, naturally attenuated strains, or other attenuation strategies allows limited viral replication and antigen exposure without causing severe disease. This induces ASFV-specific humoral and cellular immune responses, which may contribute to protection against subsequent challenge with virulent ASFV, including reduced disease severity, viremia, and improved survival (57, 58).

At the same time, promising pig challenge data do not automatically translate into routine global field deployment. Questions remain regarding biosafety, breadth of protection, DIVA compatibility, reversion risk, regulatory approval, and the extent to which country-specific or regional use should be generalized (14, 22). The baseline immunological and hygienic status of pigs may also influence ASFV infection outcomes and vaccine performance. Radulovic et al. (61) demonstrated distinct responses between specific pathogen-free and conventionally farm-raised pigs, indicating that results obtained under controlled laboratory conditions may not fully reflect those observed under field conditions. A meta-review must therefore resist both dismissal and overstatement: ASF vaccines are one of the most important areas of progress, but they are not yet equivalent to a universally mature field tool.

For SI, the situation is more operationally advanced. Whole inactivated virus vaccines are already used in commercial systems and can reduce clinical disease and viral shedding when vaccine strains are reasonably matched to circulating viruses (6, 56) (Figure 3). Important limitations include strain specificity, antigenic mismatch, maternal antibody interference in piglets, and the possibility of vaccine-associated enhanced respiratory disease under certain mismatch conditions (55, 62). Thus, SI vaccination is better understood as a necessary but imperfect component of respiratory disease management rather than as a complete solution (Table 2).

**Figure 3:**
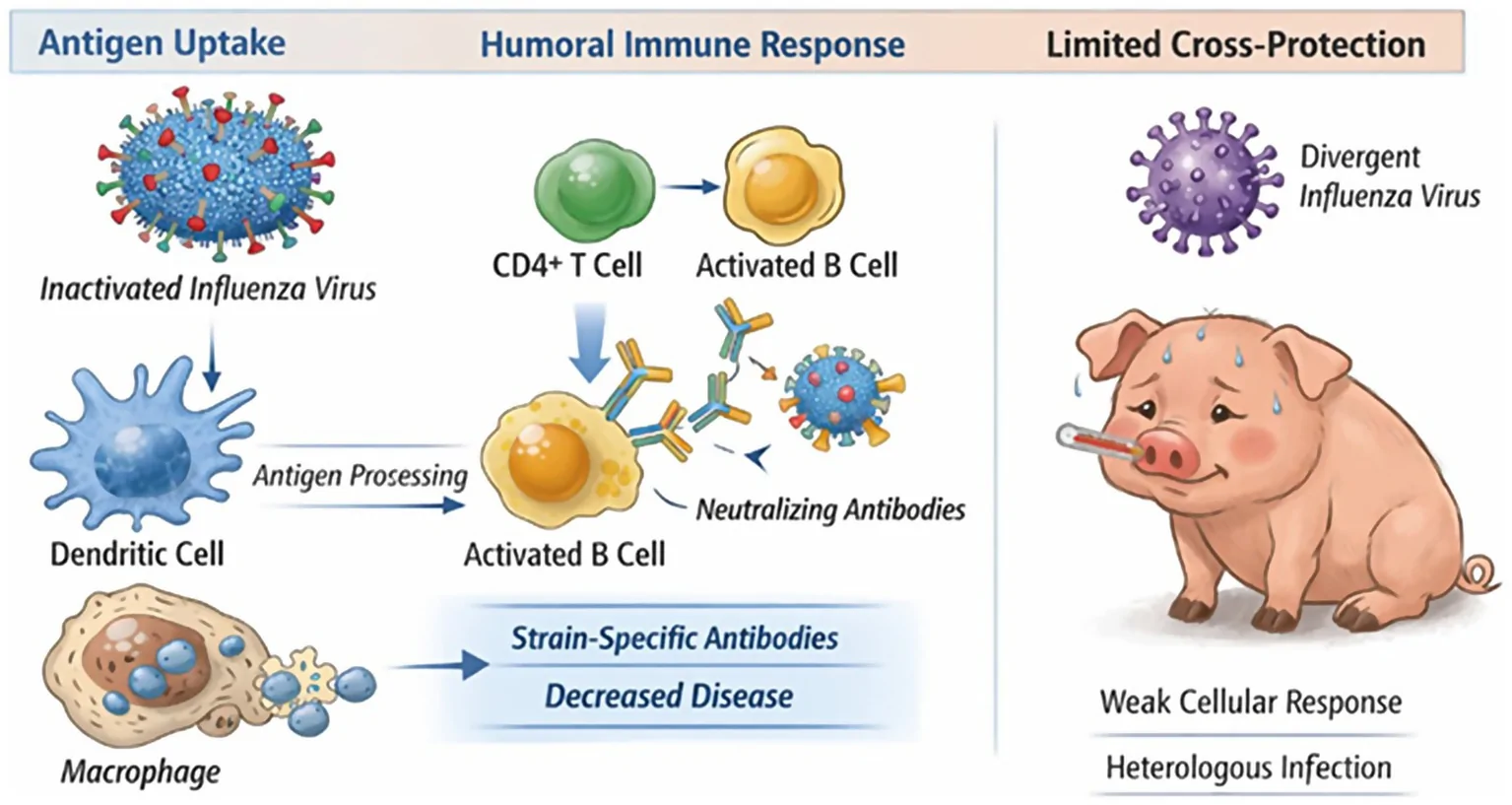
Mechanism of protection induced by whole inactivated virus (WIV) vaccines. Inactivated influenza virions are taken up by antigen-presenting cells and processed without viral replication, inducing predominantly strain-specific humoral immune responses. Vaccine-induced antibodies reduce viral replication and disease following homologous challenge but provide limited cellular immunity and cross protection.

## 7 Supportive care, environmental optimization, and nutritional support

Supportive care has to be interpreted differently in ASF and SI. In SI, supportive management is clinically meaningful because many outbreaks involve severe herd morbidity but comparatively low mortality, allowing intervention to reduce the burden of respiratory compromise, stress, and secondary bacterial disease (31). Ventilation, control of dust and ammonia, temperature stability, hydration, access to balanced feed, and veterinary management of bacterial complications can all influence recovery and production losses.

These measures are particularly relevant because respiratory viruses do not act in isolation from the production environment. Stress physiology, air quality, stocking density, and mucosal health all influence how pigs tolerate and recover from infection (20, 21). The literature cited in the source review also supports the general contribution of micronutrients and adequate nutritional status to immune function and barrier maintenance (63). In this sense, supportive care is not a direct antiviral strategy, but it is a meaningful resilience-supportive layer in SI management.

In ASF, the role of supportive care is far more limited. Acute outbreaks are characterized by severe systemic disease, rapid transmission, and high mortality such that hydration, nutrition, or general supportive measures rarely change the herd-level trajectory (8, 46). A more accurate formulation is that good husbandry supports general health in ASF-risk regions, but it should not be presented as a meaningful substitute for outbreak detection, movement restriction, carcass management, and stamping-out policies (11, 64).

## 8 Biosecurity and herd management as population-level immune regulation

Biosecurity is the most broadly applicable and practically important intervention across both diseases, and it remains the primary control pillar for ASF. The cited literature consistently supports movement control, perimeter security, cleaning and disinfection, quarantine of new animals, farm-specific clothing and equipment, wildlife exclusion, and all-in/all-out systems as high-value measures for reducing infection pressure (11, 12, 24). These measures are not merely procedural; they are epidemiological barriers that change whether pathogens reach susceptible pigs at all.

In ASF, biosecurity is essential because the virus can enter through multiple indirect routes, survive in the environment, and spread catastrophically once introduced (22, 23). In SI, biosecurity functions somewhat differently: it reduces frequency of viral introduction, decreases mixing between infected and susceptible cohorts, and limits the opportunities for persistent herd circulation and reassortment (29, 37). Thus, although the specific routes of risk differ, herd management remains central in both diseases.

The concept of immune resilience becomes most concrete at this herd level. Reduced infection pressure preserves immune competence by preventing repeated subclinical challenge, minimizing chronic inflammatory burden, and lowering the probability that animals encounter multiple overlapping pathogens during vulnerable periods such as weaning, transport, or mixing (20, 65). From this perspective, biosecurity is not external to immunology but one of the main determinants of whether a herd can maintain immune function without being overwhelmed by exposure load.

## 9 Host-directed and regenerative concepts

An emerging area of mechanistic and translational research focuses on host-directed and regenerative interventions with potential therapeutic relevance in swine, including mitochondria-targeted strategies, thymic or organ-derived peptides, and stem cell therapies (66). These approaches proposed to enhance host resilience, immune balance, mitochondrial function and tissue repair. Although these interventions have been explored in other biological contexts, their disease-specific application to ASF and SI in pigs remains unstudied. Therefore, the present review discusses these interventions based on available mechanistic, *in vitro*, and indirect *in vivo* evidence from other experimental models, while acknowledging that the evidence remains preliminary and therapeutic efficacy in pigs remains to be established. Within this framework three convergent regenerative approaches, including Mito Organelles (MO), precursor stem/progenitor cell (PSC) and nano-organo peptide (NOP) therapies are three hypothesis-generating regenerative approaches. Their potential relevance is based on mechanistic overlap with biological processes involved in ASF and SI, including mitochondrial dysfunction, oxidative stress, immune dysregulation, inflammation and impaired tissue repair are contributing factors to disease severity and recovery. Collectively, these approaches represent avenues for further investigation to establish their potential role in supporting immune resilience and recovery in swine.

### 9.1 Mitochondria-targeted strategies

Mitochondria play central roles in bioenergetics, redox regulation, innate immune signaling, and cell fate decisions, making them a credible mechanistic site of virus-host interaction. For ASFV, emerging mechanistic studies demonstrate direct manipulation of mitochondrial pathways. The ASFV MGF360-16R protein can localize to mitochondria in infected porcine macrophages and promote BAX-dependent mitochondrial apoptosis, while other viral mechanisms have been linked to lipid peroxidation, ferroptosis, and suppression of antiviral cGAS-STING signaling (67, 68). These findings indicate that mitochondrial integrity and redox homeostasis are directly involved in ASFV-host interactions. In SI, oxidative stress, mitochondrial disturbance, apoptosis and inflammatory lung injury similarly provide a mechanistic rationale for investigating strategies that preserve cellular bioenergetics and redox balance.

However, current evidence does not justify a strong translational claim in swine production. There are no robust pig efficacy data demonstrating reduced ASF mortality, reduced SI transmission, or clinically meaningful herd-level benefit from such therapy. Overall, mitochondria-targeted strategies are conceptually promising, hypothesis-generating and worthy of further investigation particularly in swine sector related to ASF and SI therapies, especially where immune-metabolic dysfunction and oxidative injury lead to disease progression.

Beyond the emerging regulatory framework for mitochondria-targeted strategy, Mito Organelle (MO) therapy is proposed as a regenerative and immune-metabolic strategy. This approach utilized cell-derived extracts containing fractionated mitochondrial proteins and peptides designed to mimic mitochondrial metabolic characteristics by engaging mechanisms analogous to bioactive peptides such as MOTS-c, Humanin and small Humanin-like peptides (69). Preliminary *in vitro* safety assessments using MTT and BALB/c-3 T3 two-stage cell transformation assays have reported no cytotoxic or carcinogenic effects, suggesting a favorable early biocompatibility profile for MO therapy (70). Furthermore, findings from the Non-Obese Diabetic (NOD) mouse model of type 1 Diabetes Mellitus showed that twice-weekly subcutaneous administration of MO peptides attenuated hyperglycemia and modulated cytokine profiles. These reflect MO peptides potential in influence immune-metabolic crosstalk and mitochondrial function—pathways relevant to ASF and SI disease (71). Although further mechanistic, target-species and clinical validations is required, these findings support continued exploration of MO therapy for its potential role to enhance immune resilience and metabolic regulation for disease management in swine (Figure 4).

**Figure 4:**
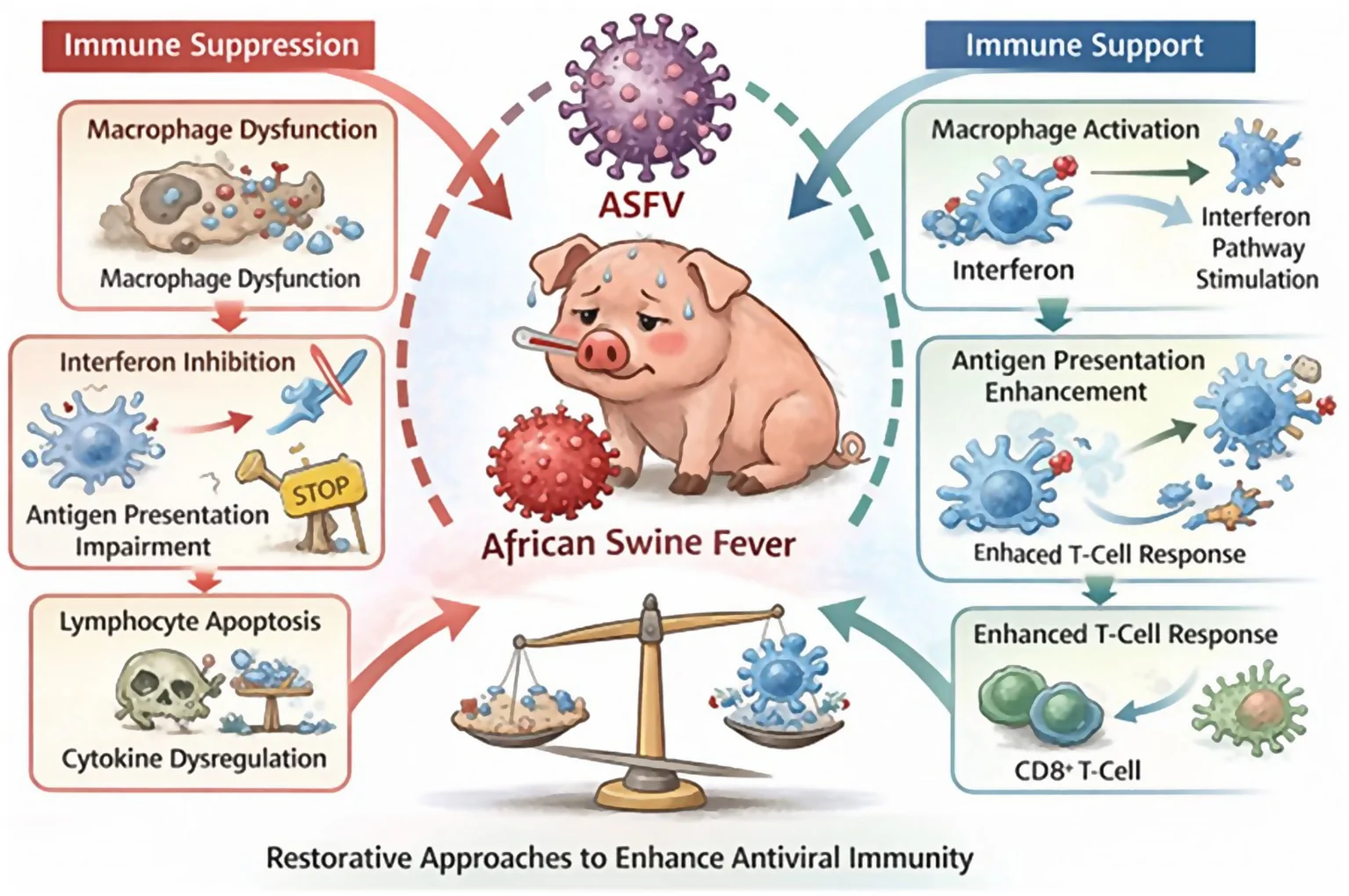
Conceptual model of ASFV-induced immune suppression and potential immune-supportive strategies. ASF infection impairs macrophage’s function, inhibits interferon signaling, disrupts antigen presentation, including lymphocyte apoptosis, leading to immune suppression. Proposed immune-support approaches aim to restore macrophage function, enhance interferon pathways, improve antigen presentation, and support T-cell responses to rebalance antiviral immunity.

### 9.2 Peptide-based strategies

Peptide-based interventions must be subdivided because the evidence is heterogeneous. Endogenous host-defense peptides, particularly porcine β-defensins, have direct experimental relevance to SI. Porcine β-defensin 2 (PBD-2) overexpression in pigs has been shown to alleviate clinical signs following swine influenza virus infection and reduce viral titres in lung tissues compared with wild-type pigs. Mechanistic studies further suggest that PBD-2 may interfere with viral infection and attenuate SI-induced cellular apoptosis (16). These findings provide disease-specific proof-of-concept for host-defense peptides in SI, although translation into a scalable therapeutic or prophylactic product remains unresolved. Equivalent evidence is currently lacking for ASFV, and peptide-based regenerative or immunomodulatory approaches have not been demonstrated to reduce ASFV replication, mortality, or transmission in pigs.

Therefore, thymic peptides and organ-derived peptide formulations are more speculative in the present disease context. Nano-organo peptide (NOP) therapy is considered an organ-specific, animal-derived peptide approach utilizing low-molecular-weight bioactive peptide fractions in a 3 nm format with a molecular-weight less than 10 kDa to enhance solubility, diffusion, receptor accessibility and tissue permeability (72). They may be biologically plausible as immunomodulatory, metabolic, regenerative, or developmental support strategies. Supportive evidence from non-ASF and non-SI models suggests that biologically derived peptide complexes can affect cellular function and regenerative pathways. For example, an isolated bioactive peptide complex derived from bovine serum with molecular weights of approximately 1,151–1,448 Da, was shown to stimulate mesenchymal stromal cell proliferation *in vitro* and enhance bone tissue regeneration *in vivo*, supporting the regenerative potential of peptide-based bioregulators (73, 74). In addition, case-based evidence reported by Chan et al. in equine wound-healing models suggests that NOP therapy may be associated with enhanced cellular turnover, angiogenesis and extracellular matrix remodeling, with observed improvements in tissue regeneration and reductions in inflammation and hypergranulation (75). However, the current literature does not establish clear ASF- or SI-specific outcome benefits in commercial swine systems. These approaches should therefore be described as conceptual or early-stage rather than as emerging practice. Collectively, these findings suggest that NOP therapy may promote regenerative and cellular regulatory pathways; however, its relevance specifically to swine herd-level disease management remains to be validated.

### 9.3 Stem cells, extracellular vesicles, and progenitor-cell products

Stem cell and extracellular vesicle strategies occupy a similar position. Their strongest biological justification lies in paracrine immunomodulation, anti-inflammatory signaling, and support of tissue repair (76, 77). In a porcine influenza model, extracellular vesicles derived from swine bone marrow mesenchymal stromal cells demonstrated antiviral and immunomodulatory activity and attenuated influenza-induced acute lung injury, providing direct proof-of-concept in the target species (78). Yet those findings remain indirect relative to the central question of whether such products can function as realistic ASF or SI interventions in production herds.

The translational barriers are substantial. These include sourcing, sterility, cold-chain logistics, lot consistency, administration route, dosing, cost, regulation, and food-chain considerations. When these constraints are considered alongside the limited disease-specific evidence, the appropriate meta-review conclusion is that regenerative biologics remain future research concepts rather than near-term tools in commercial swine medicine.

In regenerative medicine, precursor stem cells (PSC)-based approaches involve targeted organ-specific precursor stem cells, a subset of unipotent cells that may support tissue repair, paracrine signaling and modulation of inflammatory responses through xenotransplantation (69). In neural progenitor-based strategies, Moretti et al. reported findings from *in vitro* neural cell systems and *in vivo* neural injury models suggesting that neural stem cell (NSC)-derived extracellular vesicles (EVs) may provide an additional biologic modality capable of modulating mitochondrial function and cellular metabolism (79). These effects are driven by EV-associated bioactive molecules that reduce oxidative stress and promote neuronal survival. In addition, Chan et al. demonstrated in an experimental BALB/c-3 T3 cell transformation assay that xenogeneic precursor/progenitor stem cells from multiple tissue lineages did not exhibit carcinogenic or tumor-promoting activity, supporting their preliminary safety profile for potential clinical applications (69). However, their specific relevance to disease control at the swine herd level requires further validation.

## 10 Evidence hierarchy and comparative interpretation

The most useful contribution of a meta-review is not simply to list interventions, but to show how strongly each one is supported and under what conditions it is relevant. When the literature is organized in this way, a clear pattern emerges. Vaccination, biosecurity, surveillance, and herd management carry the strongest evidentiary and practical weight. Supportive care and environmental optimization are important but context-dependent, especially for SI. Nutritional and barrier-supportive concepts are reasonable adjuncts but not substitutes for disease-specific control. Host-directed biologic approaches continue to be beneficial as complementary resilience-oriented strategies with potential applications in immune modulation, tissue recovery, and supportive swine health management (Table 3).

**Table 3:** Evidence hierarchy for therapeutic and preventive approaches in swine.

Intervention class	Mechanistic rationale	*In vitro*/mechanistic support	Pig *in vivo* support	Field evidence	Practical readiness	Main limitations
Gene-deleted live attenuated ASF vaccines	Strong	Strong	Strong for homologous protection	Limited and region-specific	Moderate	Safety, regulation, deployment heterogeneity, breadth still under evaluation
Whole inactivated SI vaccines	Strong	Strong	Strong	Strong	High	Antigenic mismatch, maternal antibodies, VAERD in some contexts
Biosecurity and herd management	Strong population-level rationale	Not a laboratory category	Strong observational support	Strong	Very high	Compliance, infrastructure, cost, behavioral consistency
Supportive care and environmental optimization	Strong for SI, weak as ASF outbreak therapy	Moderate	Moderate for SI-supportive outcomes	Strong routine use in SI management	High	Limited direct antiviral effect and limited utility in acute ASF outbreaks
β-defensin-related approaches	Strong biological plausibility	Strong	Moderate, preclinical	None	Low	Translation gap and uncertain farm-scale delivery strategy
Thymic / organ-derived peptides	Plausible but indirect	Limited to moderate	Weak or indirect for ASF/SI-specific outcomes	None	Very low	Disease-specific validation lacking, risk of overinterpretation
Mitochondria-targeted peptides	Conceptually strong	Moderate to strong in mechanistic systems	Weak for ASF/SI pigs	None	Very low	No robust pig efficacy data, delivery and safety unresolved
Stem cells / EVs / progenitor-cell products	Conceptually plausible for repair and immunomodulation	Strong in broader regenerative literature	Weak to moderate indirect pig-model support	None	Very low	Cost, logistics, regulation, scalability, and disease-specific evidence gaps

## 11 Integrated resilience framework

A layered model of resilience can be used to integrate the diverse intervention strategies described in the literature. In the outer layer, surveillance and biosecurity determine whether pathogens reach the herd and how rapidly their presence is recognized (42, 44). In the middle layer, vaccination, herd structure, and animal flow influence transmission intensity, clinical burden, and the extent of repeated exposure (3, 37). In the inner layer, resilience depends on nutritional status, environmental load, stress physiology, mucosal integrity, and possibly, in the future, targeted host-directed interventions (20, 21, 63).

Within this framework, immune resilience is not best understood as a competing therapeutic platform but as an emergent property of reduced infection pressure, preserved immune function, controlled inflammation, and supported recovery within a managed production system (3). This perspective highlights that the most effective resilience interventions are often those that reliably limit pathogen burden while maintaining physiological capacity for recovery.

## 12 Discussion

The comparative framing of ASF and SI provides a useful perspective for understanding how differences in pathogen biology influence disease control. ASF depends heavily on exclusion, early detection, biosecurity, surveillance, movement control and rapid containment because of its high mortality and environmental or wildlife-associated persistence. In contrast, SI is generally managed as an endemic respiratory disease through vaccination, herd immunity, strain surveillance and transmission control, although antigenic drift, strain mismatch and maternal antibodies can reduce vaccine effectiveness (27, 29, 30).

Vaccination further illustrates this contrast. SI vaccination is already established in commercial practice, whereas ASF vaccination remains less mature and is limited by concerns regarding safety, cross-protection, DIVA capability, viral diversity and field consistency (14, 22). Therefore, ASF vaccination should complement, rather than replace, biosecurity and outbreak-control measures.

Despite these differences, host factors influence disease outcomes in both infections. Immune competence, inflammatory regulation, age, co-infections, metabolic status and environmental stress can affect disease severity (80). Immune resilience should therefore be viewed as the ability to mount an effective antiviral response while limiting excessive inflammation and preserving tissue function. In ASF, host-directed approaches are more relevant as complementary research strategies for immune dysregulation, disease tolerance and recovery, whereas in SI, improving mucosal immunity and controlling pulmonary inflammation may more directly reduce disease severity and production losses (46).

Important gaps remain in the development of immune-resilience strategies for ASF and SI. ASF research requires clearer protective immune correlates across viral genotypes and vaccine platforms, as well as improved understanding of immune evasion and disease tolerance, while SI research must address antigenic evolution, reassortment and the need for broader, more durable mucosal immunity. Future studies should validate interventions under commercial production conditions using standardized characterization, dose–response studies, controlled challenge experiments and independent replication, particularly for host-directed and regenerative approaches such as MO, PSC and NOP, whose efficacy in pigs remains unconfirmed. These strategies should be evaluated as complements to vaccination, biosecurity and antiviral control using standardized virological, immunological, clinical and production-related outcomes.

## 13 Conclusion

African swine fever and swine influenza remain highly appropriate diseases for comparative review because they reveal two different but interconnected dimensions of vulnerability in global swine health. ASF is defined by lethality, environmental persistence, and the central importance of preventing introduction and limiting spread after incursion (22, 64). SI is defined by high transmissibility, recurrent productivity losses, zoonotic significance, and the challenge of maintaining vaccine effectiveness in the face of viral evolution (6, 43). These differences justify different operational priorities but also support a shared resilience framework.

On current evidence, the core pillars of control remain surveillance, biosecurity, herd management, and vaccination where biologically and operationally feasible (11, 12). Supportive care, environmental optimization, and nutritional adequacy are meaningful adjuncts, especially for SI, but they do not replace pathogen-specific control (10, 63). Host-directed peptides, mitochondrial strategies, and regenerative biologics are emerging areas of investigation that may contribute to resilience-oriented approaches, although their role in large-scale production systems continues to evolve alongside the evidence base (69, 79, 81).
